# Supplementary material for: Causal Relationship between Aging and Anorexia Nervosa: A White-Matter-Microstructure-Mediated Mendelian Randomization Analysis
Source: Biomedicines. 2024 Aug 16;12(8):1874. doi: 10.3390/biomedicines12081874 (PMC11351342; doi:10.3390/biomedicines12081874)
Supplement: Supplementary file 1 [file biomedicines-12-01874-s001.zip › Supplementary Figure/Figure S4/Singe SNP plot of MR analysis for TL to ubm-b-1645.pdf]

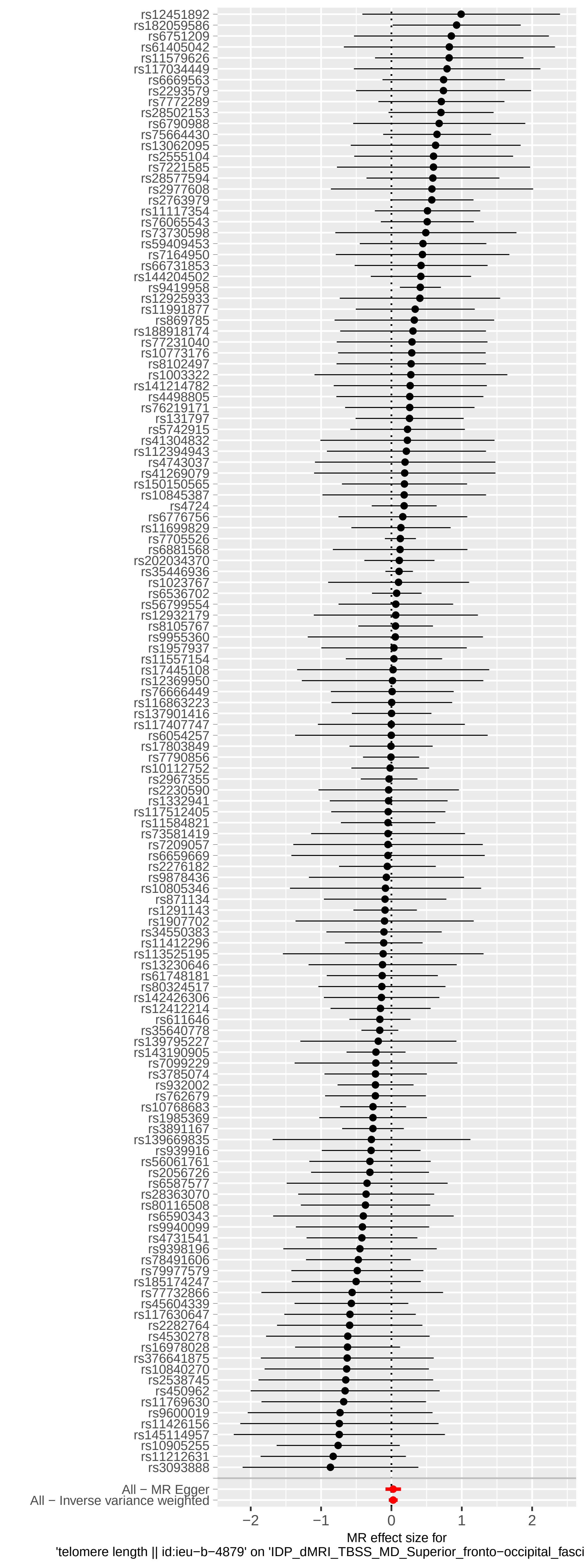
rs12451892
rs182059586
rs6751209
rs61405042
rs11579626
rs117034449
rs6669563
rs2293579
rs7772289
rs28502153
rs6790988
rs75664430
rs13062095
rs2555104
rs7221585
rs28577594
rs2977608
rs2763979
rs11117354
rs76065543
rs73730598
rs59409453
rs7164950
rs66731853
rs144204502
rs9419958
rs12925933
rs11991877
rs869785
rs188918174
rs77231040
rs10773176
rs8102497
rs1003322
rs141214782
rs4498805
rs76219171
rs131797
rs5742915
rs41304832
rs112394943
rs4743037
rs41269079
rs150150565
rs10845387
rs4724
rs6776756
rs11699829
rs7705526
rs6881568
rs202034370
rs35446936
rs1023767
rs6536702
rs56799554
rs12932179
rs8105767
rs9955360
rs1957937
rs11557154
rs17445108
rs12369950
rs76666449
rs116863223
rs137901416
rs117407747
rs6054257
rs17803849
rs7790856
rs10112752
rs2967355
rs2230590
rs1332941
rs117512405
rs11584821
rs73581419
rs7209057
rs6659669
rs2276182
rs9878436
rs10805346
rs871134
rs1291143
rs1907702
rs34550383
rs11412296
rs113525195
rs13230646
rs61748181
rs80324517
rs142426306
rs12412214
rs611646
rs35640778
rs139795227
rs143190905
rs7099229
rs3785074
rs932002
rs762679
rs10768683
rs1985369
rs3891167
rs139669835
rs939916
rs56061761
rs2056726
rs6587577
rs28363070
rs80116508
rs6590343
rs9940099
rs4731541
rs9398196
rs78491606
rs79977579
rs185174247
rs77732866
rs45604339
rs117630647
rs2282764
rs4530278
rs16978028
rs376641875
rs10840270
rs2538745
rs450962
rs11769630
rs9600019
rs11426156
rs145114957
rs10905255
rs11212631
rs3093888
All − MR Egger
All − Inverse variance weighted
−2
−1
0
1
2
MR effect size for
'telomere length || id:ieu−b−4879' on 'IDP_dMRI_TBSS_MD_Superior_fronto−occipital_fasci
